# *GJA1* dysfunction suppresses Wnt/c-MYC signaling and promotes inflammatory imbalance and MMP3-associated ECM remodeling in fibroblasts and mesenchymal stromal cells from patients with ODDD

**DOI:** 10.3389/fcell.2026.1959966

**Published:** 2026-10-16

**Authors:** Siyuan Tao, Binshan Zhao, Xiaodong Wang, Xiaofang Shen, Xiuli Zhao

**Affiliations:** 1 Center for Rare Diseases, State Key Laboratory of Complex, Severe, and Rare Diseases, Peking Union Medical College Hospital, Chinese Academy of Medical Sciences & Peking Union Medical College, Beijing, China; 2 Key Laboratory in Science and Technology Development Project of Suzhou (CN), Pediatric Orthopedics, Children’s Hospital of Soochow University, Suzhou, China; 3 Department of Medical Genetics, State Key Laboratory for Complex, Severe, and Rare Diseases, Institute of Basic Medical Sciences, Chinese Academy of Medical Sciences, School of Basic Medicine, Peking Union Medical College, Beijing, China

**Keywords:** Cx43, GJA1, NF-κB, oculodentodigital dysplasia (ODDD), Wnt/β-catenin

## Abstract

Oculodentodigital dysplasia (ODDD) is caused by variants in *GJA1*, but its downstream molecular network remains unclear. This study found that *GJA1* defects lead to comprehensive suppression of Wnt/β-catenin signaling and the co-activator c-MYC in patients’ skin fibroblasts and mesenchymal stem cells (MSCs). This change induces a dual pathological process: On the one hand, the loss of Wnt lifts the transcriptional repression of *MMP3*, leading to abnormally high secretion of matrix metalloproteinases; on the other hand, the absence of *c-MYC* results in a specific inflammatory bias in NF-κB activation (a surge in *IL-6*, while *CXCL8* (*IL-8*)are paradoxically and significantly reduced), accompanied by impaired cell migration, proliferation, and osteogenic differentiation. To validate the causal relationship, we demonstrated through genetic intervention that knocking down *GJA1* in normal cells replicates the patient phenotype, while overexpressing wild-type *GJA1* completely reverses the aforementioned abnormalities. Pharmacological intervention further revealed the hierarchical relationships within the pathway: the Wnt activator (CHIR99021) targets a core upstream hub, correcting *CXCL8* abnormalities, inhibiting *MMP3*, and promoting osteogenesis by restoring *c-MYC*; in contrast, simply blocking IL-6 (IL6i) acts only on downstream effects; although it can downregulate the interferon response, it fails to restore *c-MYC* and thus cannot rescue *CXCL8* or effectively inhibit *MMP3*; the combination of the two produces the optimal combined effect. Collectively, we define a novel pathogenic framework linking *GJA1* dysfunction to Wnt/c-MYC suppression-driven inflammatory bias and ECM degradation, identifying Wnt activation as a potential therapeutic avenue requiring further validation.

## Introduction

Oculodentodigital dysplasia (ODDD) is a rare autosomal dominant disorder caused by germline variants in *GJA1*, which encodes the gap junction protein connexin 43 (Cx43) (Paznekas et al., 2003; Laird, 2014). Patients with ODDD typically exhibit a pleiotropic phenotype affecting the eyes, teeth, digits, and central nervous system (Loddenkempe et al., 2002; Paznekas et al., 2009). ODDD phenotypic severity varies widely. Genotype–phenotype correlations remain incompletely defined, although C-terminal domain variants are often associated with neurological involvement. *GJA1* encodes Cx43, and these variants result in dysfunctional Cx43, which is the molecular basis of ODDD. Cx43 is the most ubiquitously expressed connexin and plays critical roles in various tissues by forming gap junction channels (Söhl and Willecke, 2004). Structurally, Cx43 comprises four transmembrane domains, an intracellular loop, and a long C-terminal tail that interacts with multiple signaling molecules (Nambara et al., 2007). Beyond channel-dependent functions, accumulating evidence indicates that Cx43 also exerts channel-independent functions via its C-terminal domain, which associates with signaling partners including β-catenin and various kinases, thereby modulating cellular homeostasis and signal transduction (Dbouk et al., 2009; Cai et al., 2026).

Previous studies using heterologous expression systems have demonstrated that ODDD-linked Cx43 variants often exhibit impaired trafficking, intracellular retention, and dominant-negative effects on wild-type Cx43 (Gong et al., 2007). Patient-derived dermal fibroblasts show reduced proliferation, impaired migration, and altered extracellular matrix (ECM) remodeling (Churko et al., 2011; Kelly et al., 2016; Esseltine et al., 2015), while ODDD iPSCs exhibit delayed osteogenic and chondrogenic differentiation (Esseltine et al., 2017). Previous studies have established roles for Cx43 in skeletal biology and have described crosstalk between Wnt/β-catenin and NF-κB signaling (Ma and Hottiger, 2016; Stains and Civitelli, 2016). However, whether *GJA1* dysfunction links these pathways in ODDD patient-derived cells remains unclear.

Wnt/β-catenin signaling is a master regulator of developmental processes and cell fate determination, and its dysregulation is often accompanied by aberrant activation of inflammatory pathways such as NF-κB (Clevers, 2006; Logan and Nusse, 2004). In ODDD, although *GJA1* variants are known to cause pleiotropic defects, the downstream signaling network—particularly the interplay between Wnt suppression and inflammatory responses—has remained largely unexplored. Critically, no previous study has uncovered the c-MYC dependent paradoxical inflammatory imbalance characterized by concurrent IL-6 overproduction and CXCL8 suppression in ODDD. Moreover, most prior work relied on heterologous systems or iPSCs, whereas primary patient fibroblasts and mesenchymal stromal cells (MSCs) carry native pathogenic variants and better recapitulate patient pathophysiology, offering superior translational relevance.

To address these gaps, we designed two core objectives: (1) to delineate the complete molecular network through which *GJA1* variants disrupt Wnt/c-MYC signaling, driving inflammatory bias, excessive ECM remodeling, and dysregulated mesenchymal lineage differentiation in primary patient-derived cells; and (2) to clarify the hierarchical order of downstream signaling cascades via complementary genetic and pharmacological interventions, thereby identifying actionable therapeutic targets. Our findings demonstrate that restoring the Wnt/c-MYC axis, rather than blocking isolated downstream inflammatory cytokines, comprehensively rescues ODDD-associated cellular defects, highlighting Wnt activation as a promising therapeutic strategy for ODDD.

## Materials and methods

### Subjects and DNA samples

Two ODDD patients (P1 and P2), their available family members, and three healthy controls were all recruited from the Department of Pediatric Orthopedics, Children’s Hospital of Soochow University (Suzhou, China) between December 2023 and January 2026. The age and sex of all participants are summarized in Table 1 and Supplementary Table S1. Diagnosis of ODDD was based on at least two major clinical criteria (facial dysmorphism, syndactyly, dental anomalies), and was confirmed by clinical examination, X-ray imaging, and Sanger sequencing of *GJA1*. Peripheral blood samples and discarded surgical bone and skin tissues were collected from the ODDD patients during orthopedic procedures (syndactyly release or corrective osteotomy of the hand/digit). Peripheral blood samples were obtained from available family members. For healthy controls, peripheral blood and discarded surgical bone and skin tissues were collected from age and sex-matched pediatric patients undergoing hand trauma surgery at the same anatomical site; these controls had no genetic or inflammatory disorders, and whole-genome sequencing was performed to exclude pathogenic variants in known disease-associated genes. Patient and control samples were matched for age, sex, anatomical site, and clinical context. Blood samples (3–5 mL) were collected from all available family members, and genomic DNA was extracted using the standard sodium dodecyl sulfate–proteinase K-phenol/chloroform method.

### Cell culture

With informed consent, we aseptically collected human bone and skin tissues as discarded surgical specimens during clinically indicated orthopedic procedures. Patient samples were collected during syndactyly release or corrective osteotomy of the hand/digit, and control samples were obtained from age- and sex-matched pediatric patients undergoing hand trauma surgery at the same anatomical site. All experiments used cells at passage 3–5. Supplementary Table S1 provides detailed donor information. For MSCs, tissues were minced into 1 mm^3^ pieces, washed in Phosphate-Buffered Saline (PBS) plus antibiotics, and sequentially digested in 0.25% trypsin (37 °C, 20 min) and 2 mg/mL collagenase (37 °C, 2 h). Digested products were filtered (40 μm), centrifuged (1,000 rpm, 5 min), and then cell pellets were cultured in low-glucose Dulbecco’s modified Eagle’s medium (DMEM) supplemented with 10% fetal bovine serum and 100 μg/mL penicillin-streptomycin at 37 °C in a 5% CO_2_ incubator until they reached 80%–90% confluence. The remaining bone fragments were also cultured. For fibroblasts, tissue was minced, washed, placed in a T25 flask (cross-sections facing up), incubated upright overnight in serum-free medium, and cultured in Dulbecco’s Modified Eagle Medium/Nutrient Mixture F-12 (DF-12) supplemented with 10% fetal bovine serum and 100 μg/mL penicillin-streptomycinat 37 °C in a 5% CO_2_ incubator until they reached 80%–90% confluence. Low-glucose DMEM was used for bone-derived MSCs to maintain stemness and promote adherence, whereas DF-12 was used for skin fibroblasts to support outgrowth from small tissue explants.

### Flow cytometric characterization of MSCs

MSCs at passage 3–5 were harvested and resuspended in PBS. Cells were incubated with fluorophore-conjugated anti-human antibodies for 30 min at 4 °C in the dark: CD90-FITC (Invitrogen, 11–0,909–42), CD105-FITC (Invitrogen, MA5-46642), CD73-FITC (Invitrogen, 17–0,739–42), and CD45-FITC (Invitrogen, 58–0,459–42). Matched isotype antibodies were used as negative controls. After washing, samples were analyzed on a flow cytometer, and data were processed using FlowJo software (v10.8). Cells were considered MSCs if they were positive for CD90, CD105, and CD73 (≥95%) and negative for CD45 (≤2%).

### Whole-genome sequencing

Genomic DNA was randomly fragmented to approximately 350 bp using the Covaris fragment. Subsequent end repair and end-blunting ensured 5′-end phosphorylation. The repaired products were purified using Agencourt SPRIselect magnetic beads and subjected to fragment selection with the Agencourt SPRIselect nucleic acid fragment selection kit to enrich target-length fragments. Purified, end-blunted DNA fragments underwent A-tailing to enable ligation with T-overhang sequencing adapters. The adapter-ligated library was PCR-amplified for enrichment, followed by quality control. Based on effective library concentration and data volume requirements, final sequencing was performed on the Illumina Nova Seq 6,000 platform.

### PCR and sanger sequencing

Candidate variants identified through WGS were validated using PCR-Sanger sequencing, and family-based linkage analysis was performed to assess phenotypic-genotypic linkage. Reference sequences for genomic DNA/cDNA and protein (hg19: NM_000165.5 and NP_000156) of the *GJA1* gene were obtained from the UCSC Genome Browser (http://genome.ucsc.edu/) and the NCBI Reference Sequence Database. Primers were designed using the Primer3 online tool (http://primer3.ut.ee/) and validated with BLAT and the online virtual PCR tool from the UCSC Genome Browser (Supplementary Table S2). All PCR reactions employed TaKaRa LA Taq® with GC Buffer (TaKaRa, Shiga, Japan). Reaction conditions were as follows: 95 °C pre-denaturation for 3 min; 35 cycles of 94 °C for 30 s, 58 °C for 40 s, and 72 °C for 40 s; followed by a final extension at 72 °C for 8 min. Sequencing was performed on an Applied Biosystems 3,730×l DNA Analyzer (Thermo Fisher Scientific, Waltham, USA). Results were aligned against reference sequences using Codon Code Aligner software (v.6.0.2.6; Codon Code, Centerville, USA).

### RNA sequencing and analysis

Total RNA was extracted from cultured skin fibroblasts derived from patients with ODDD (n = 2) and age-matched healthy controls (n = 3), as well as from MSCs derived from one ODDD patient (P1) and three healthy controls (n = 3). For each cell type and donor, RNA was extracted from three independent biological replicates (each replicate representing a separately cultured passage). RNA extraction was performed using TRIzol reagent (Life Technologies, California, USA) according to the manufacturer’s protocol. RNA concentration and purity were measured using a NanoDrop 2000 spectrophotometer (Thermo Fisher Scientific, Wilmington, DE, USA), and RNA integrity was assessed using the RNA Nano 6000 Assay Kit on an Agilent Bioanalyzer 2,100 system (Agilent Technologies, California, USA). Samples with RNA integrity number (RIN) ≥ 8.0 were used for library construction.

cDNA libraries were constructed and sequenced on an Illumina platform (NovaSeq 6,000) with paired-end 150 bp reads. Raw reads were quality-filtered and trimmed using Trimmomatic, and clean reads were aligned to the human reference genome (GRCh38) using HISAT2. Gene expression levels were quantified using featureCounts, and transcriptional pattern/candidate pathway enrichment. Gene Ontology (GO) and Kyoto Encyclopedia of Genes and Genomes (KEGG) pathway enrichment analyses were performed using clusterProfiler to annotate the functional roles of DEGs. A hypergeometric test was applied, and terms with a p-value <0.05 were considered significantly enriched.

### Osteogenic induction and adipogenic induction

MSCs were induced toward osteogenic differentiation using the OriCell® Human-Related Stem Cell Osteogenic Induction and Differentiation Kit (Cyagen Biosciences, HUXXC-90021, U.S.A) for seven or 14 days, with medium changes every 2–3 days. MSCs were induced toward adipogenic differentiation using the OriCell® Human Bone Marrow MSC adipogenic differentiation kit (Cyagen Biosciences, HUXMX-90031, U.S.A). Cells (5 × 10^4^ cells/cm^2^) were seeded in culture plates pre-coated with 0.1% gelatin (Cyagen Biosciences, GLT-11301, U.S.A). At 100% confluence, the growth medium was replaced with adipogenic induction medium A. After 3 days of induction, the medium was switched to adipogenic maintenance medium B for 1 day. This induction (Medium A) and maintenance (Medium B) cycle was repeated four times and typically lasted 14 days.

### Knockdown of GJA1 in normal cells

To precisely mimic the *GJA1* deficiency observed in ODDD patients, normal control dermal fibroblasts (FBs) and mesenchymal stem cells (MSCs) were transfected with a pre-designed *GJA1* siRNA set containing three specific siRNAs (Cat# HY-RS05440, MedChemExpress, China). Cells at 70%–80% confluence in 6-well plates were transfected with *GJA1* siRNA (si-*GJA1*) using Lipofectamine™ 3,000 (Invitrogen, USA). Cells transfected with non-targeting scrambled siRNA (si-NC) served as controls. At 48 h post-transfection, RT-qPCR was performed to confirm that GJA1 mRNA levels in normal cells were downregulated to approximately 50% of the control baseline, which corresponds to the endogenous expression levels observed in patient-derived cells.

### Overexpression of wild-type GJA1 in patient cells

To evaluate whether restoring *GJA1* expression reverses the pathological phenotypes in ODDD, patient-derived fibroblasts and MSCs were transfected with the wild-type human Cx43 expression plasmid, pcDNA3.2-Cx43-HA (Plasmid #49851, Addgene, USA), using Lipofectamine™ 3,000. Patient cells transfected with the empty vector (pcDNA3.2) served as mock controls. Transfection efficiency was validated by RT-qPCR 48 h post-transfection, confirming that *GJA1* mRNA levels in patient cells were restored to levels comparable to those of normal control cells.

### Knockdown and overexpression of c-MYC

To directly test the causal role of c-MYC, normal control MSCs were transfected with c-MYC siRNA (Santa Cruz, sc-29226) using Lipofectamine™ 3,000 for 48 h. Cells transfected with scrambled siRNA (si-NC) served as negative controls. MSCs derived from patient (P1) were transfected with pcDNA3.1-c-MYC plasmid (Addgene #46975) or empty vector for 48 h. Transfection efficiency was confirmed by RT-qPCR. *CXCL8* and *MMP3* mRNA levels were quantified as described above.

### Pharmacological treatments

To investigate the hierarchical relationship of downstream signaling cascades, patient-derived cells were subjected to pharmacological treatments. The Wnt/β-catenin pathway activator CHIR99021 (Cat# SML1046, Sigma-Aldrich, USA) was dissolved in DMSO to prepare a stock solution and applied at a final concentration of 10 μM for 24 h, with cells treated with an equal volume of DMSO (0.1% final concentration) serving as vehicle controls. Additionally, human IL-6 neutralizing antibody (Cat# 10395-MHK23, Sino Biological, China; RRID: AB_2860167) was used at a final concentration of 5 μg/mL for 24 h, with PBS containing 0.1% BSA used as a control. For combinatorial treatments, cells were simultaneously exposed to both agents for 24 h.

### RNA extraction and quantitative real-time PCR (RT-qPCR)

Total RNA was extracted from human fibroblasts, MSCs, and osteoblasts using the TRIzol method. Cells were washed with ice-cold PBS, lysed with TRIzol (Invitrogen, Waltham, MA, USA), chloroform-extracted, precipitated with isopropanol, washed with 75% ethanol (DNase/RNase-free), and finally dissolved in enzyme-free water. RNA integrity was assessed by denaturing gel electrophoresis stained with ethidium bromide. PrimeScript RT Kit (TaKaRa, Dalian, China) was used to reverse transcribe 500 ng total RNA into cDNA. RT-qPCR was performed on a Bio-Rad iCycler system using TB Green (TaKaRa, Dalian, China) dye in a 20 μL reaction volume. Amplification program: 95 °C for 30 s; 95 °C for 5 s, 58 °C for 30 s, 65 °C for 5 s, repeated 39 times; 95 °C for 5 s. *GAPDH* served as the internal control, and relative gene expression was calculated using the 2^−ΔΔCT^ method. Primers were designed using the NCBI database; specific sequences are listed in Supplementary Table S3. All experiments were repeated three times.

### Western blotting

Cells were lysed in RIPA buffer (Beyotime, #P0013C, China) supplemented with protease and phosphatase inhibitors (Beyotime, #P1091, China). Protein concentrations were determined using a BCA assay (Thermo Fisher Scientific, #23227, U.S.A). Equal amounts of protein were separated by SDS-PAGE (4%–12% gels) and transferred to PVDF membranes (Thermo Fisher Scientific, #88520, U.S.A). Membranes were blocked with 5% non-fat milk in PBST, then incubated with primary and HRP-conjugated secondary antibodies. GAPDH served as the loading control. Protein bands were visualized using enhanced chemiluminescence (ACE, #BK0042-01, China) and analyzed with Image Lab Software (Bio-Rad). See Supplementary Table S4 for antibody details.

### Immunofluorescence analysis

Cells were cultured on coverslips and subjected to immunofluorescence staining. Specific steps: Wash three times with PBS; fix with 4% paraformaldehyde at room temperature; wash with PBS; permeabilize with 0.2% Triton X-100 (Thermo Fisher Scientific, #85111, U.S.A); block with 5% BSA (Beyotime, ST025, China); incubate overnight at 4 °C with primary antibody; wash with PBS; incubate with fluorescently labeled secondary antibody at room temperature; stain nuclei with DAPI; mount with anti-fluorescence quenching mounting medium. Images were acquired and analyzed under a fluorescence microscope using the following configurations: DAPI (excitation 330–380 nm, emission 420 nm, blue light); FITC (excitation 465–495 nm, emission 515–555 nm, green light); Alexa Fluor 594 (excitation 590 nm, emission 617 nm, red light). Fluorescence intensity was quantified using ImageJ software. For each sample, at least five randomly selected high-power fields were imaged, and all DAPI-positive cells within these fields were analyzed.

### Enzyme-linked immunosorbent assay (ELISA)

To quantify the secretion levels of IL-6 and IL-8 in the culture supernatants, commercial Human IL-6 ELISA Kit (Cat# GZEH0001-48 T) and Human IL-8 ELISA Kit (Cat# GEH0005-48 T) from Servicebio (China) were utilized according to the manufacturer’s protocols. Briefly, culture supernatants were centrifuged at 1,000 × g for 10 min at 4 °C to remove cellular debris. A total of 100 μL of standards and samples were added to the pre-coated 96-well plates and incubated at 37 °C for 90 min. After washing, biotinylated antibody working solution was added and incubated for 60 min. Following another washing step, streptavidin-HRP working solution was added and incubated for 30 min. Plates were washed again, and TMB substrate solution was added and incubated in the dark for 15–20 min. The reaction was terminated with stop solution. The optical density (OD) was measured at 450 nm using a microplate reader. The absolute concentrations of IL-6 and IL-8 were extrapolated from standard curves. All samples were assayed in triplicate.

### Migration assays

Logarithmic growth phase cells were starved for 12 h in serum-free medium. After trypsin digestion, cells were washed twice in PBS, resuspended in serum-free medium, and the density adjusted to 5 × 10^4^/mL. An upper Transwell chamber was filled with 200 μL of cell suspension, and the lower chamber was filled with serum-containing medium. Cells were cultured at 37 °C for 48 h, after which the small chamber was removed. Non-migrated cells in the upper chamber were gently wiped away with a cotton swab after washing in PBS. Cells were then fixed in 4% paraformaldehyde for 15 min, followed by 0.1% crystal violet (Beyotime, C0121, China) staining for 30 min. Cells were then washed three times in PBS and photographed under a microscope (Olympus, JPN).

### 5-Ethynyl-2’-deoxyuridine (EdU) staining

Cell proliferation was assessed using the EdU-488 detection kit (Beyotime, C0071S, China). Cells cultured in 24-well plates were incubated with 20 µM EdU for 2 h, then fixed and permeabilized in 0.3% Triton X-100. After washing, a click reaction was performed in the dark for 30 min. Nuclei were stained with DAPI. Samples were then imaged under fluorescence microscopy, with Azide 488 fluorescence detected at 495 nm (excitation wavelength) and 519 nm (emission wavelength). Fluorescence intensity was quantified using ImageJ software. For each sample, at least five randomly selected high-power fields were imaged, and all DAPI-positive cells within these fields were analyzed.

### Alkaline phosphatase (ALP) and alizarin red S staining

After 7 days of osteogenic induction, cells were washed three times in PBS, fixed in 4% (v/v) paraformaldehyde, and incubated with ALP staining solution (Beyotime, #P0321, China) for 1 h in the dark. Alizarin Red S staining was performed 14 days after induction. Cells were washed three times in PBS, fixed in 4% (v/v) paraformaldehyde, and then incubated with Alizarin Red S staining solution (Beyotime, C0138, China) for 30 min.

### Oil red O staining

Following adipogenic differentiation, cells were washed in PBS and fixed in 4% paraformaldehyde. After washing, lipid droplets were stained with a freshly prepared Oil Red O working solution (Beyotime, #C0157, China) for 30 min at room temperature. Cells were then washed and counterstained with hematoxylin. Stained samples were observed by microscopy, with lipid droplets appearing red and nuclei blue.

### Quantification and statistical analysis

All experiments were performed in a blinded manner whenever possible. For control experiments, primary fibroblasts and MSCs were obtained from three independent healthy donors (biological replicates, n = 3). For patient experiments, fibroblasts were obtained from two ODDD probands (P1 and P2), whereas MSCs were obtained from only one ODDD proband (P1) because of the extreme rarity of ODDD and the limited availability of pediatric bone tissue. Each functional assay was performed using at least three independent technical replicates from different passages of the corresponding patient-derived or control cells.

For comparisons between patient-derived and control cells, unpaired two-tailed Student’s t-tests were used where appropriate, with technical replicates from patient-derived samples compared with biological replicates from three independent healthy donors. This analytical approach follows the established framework for rare disease studies (Botta et al., 2023), while acknowledging the inherent statistical limitations when independent biological replicates from patient samples are unavailable. Because ODDD is an ultra-rare disorder and patient-derived samples were limited, statistical analyses involving patient-derived cells were considered exploratory. Technical replicates from the same patient were not treated as independent biological replicates, and statistical results involving patient-derived samples should therefore be interpreted with caution.

For comparisons between two conditions within the same patient donor, paired two-tailed Student’s t-tests were used where appropriate. For comparisons involving three or more experimental groups, one-way ANOVA followed by Tukey’s multiple-comparisons test was used where appropriate.

Because only one patient-derived MSC sample was available for RNA-seq, the MSC transcriptomic analysis was considered exploratory. Gene-level differences identified from this dataset were not interpreted as statistically validated differential-expression results. Instead, the RNA-seq data were used to characterize global transcriptional patterns and identify candidate genes and pathways for subsequent experimental validation.

All data are presented as mean ± SD. Statistical significance was defined as *p < 0.05, **p < 0.01, ***p < 0.001, and ****p < 0.0001. Statistical analyses were performed using GraphPad Prism 9 (GraphPad Software, San Diego, CA, USA).

## Results

### Clinical characteristics and validation of patient-derived MSCs

Proband 1 presented with typical ODDD. She had short palpebral fissures and epicanthal folds, as well as other typical ODDD facial features, such as hypoplastic alae nasi, a prominent nasal tip and columella, and thin lips; furthermore, the patient presented with sparse hair. Limb abnormalities included bilateral syndactyly of fingers 4–5. Dental anomalies, including microdontia, dental crowding, and enamel hypoplasia, were also observed (Figure 1A). The affected younger brother of the proband presented with identical craniofacial and hand phenotypes, accompanied by sparse hair. As depicted in the pedigree (Figure 1B), the mother of Family 1 exhibited a mild phenotype, restricted solely to bilateral brachydactyly of the fifth fingers, while the father was clinically unaffected. None of the patients exhibited overt neurological deficits or other severe congenital malformations, and brain MRI was not performed due to the absence of central nervous system-related clinical manifestations. We cannot fully rule out subclinical occult central lesions, and long-term neurological follow-up is required. Intellectual development of both patients was normal.

**FIGURE 1:**
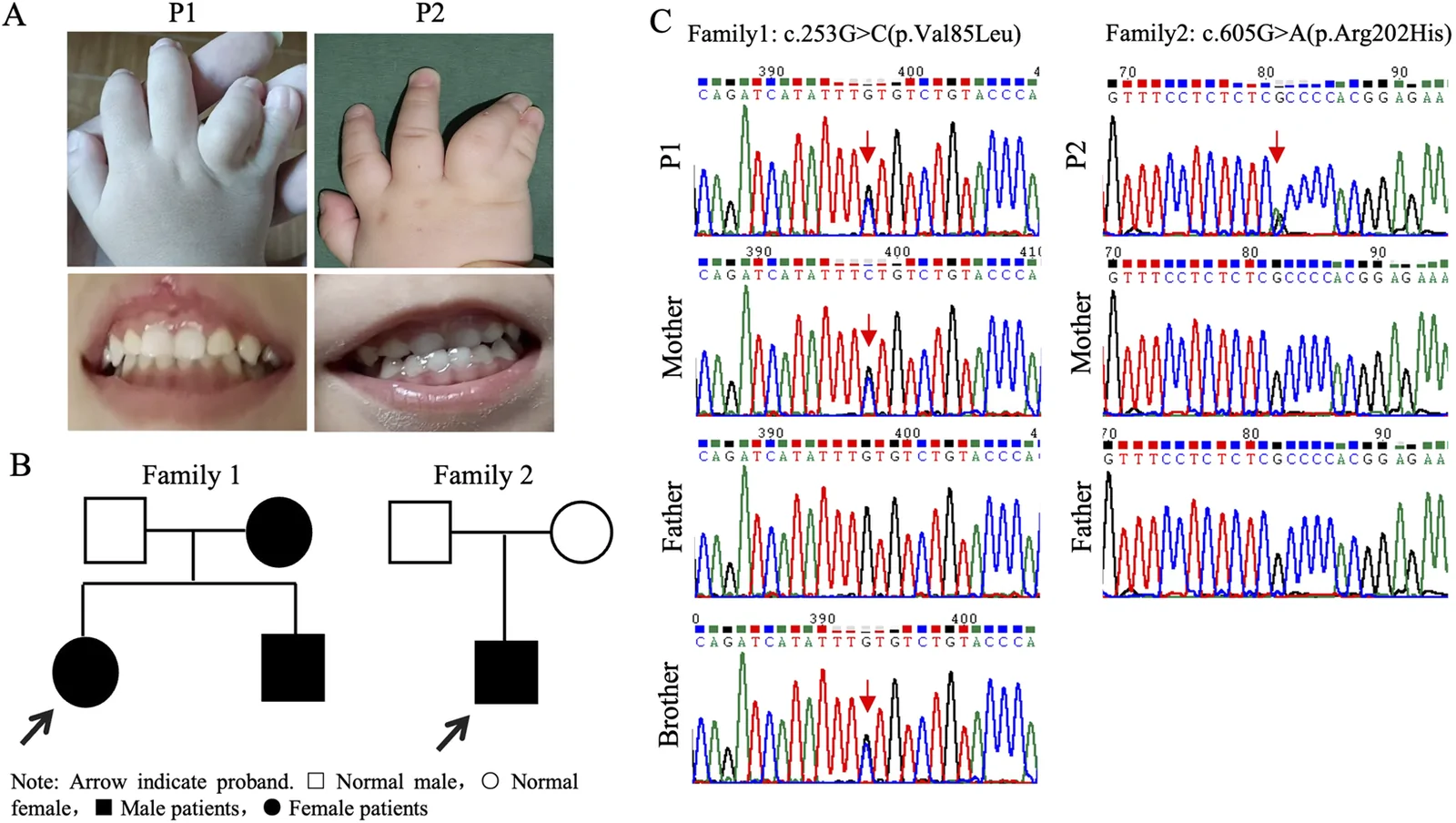
Clinical manifestations, pedigree, and pathogenic variation verification of two families. **(A)** Clinical manifestations of probands. **(B)** Pedigrees of the two families. **(C)** Sanger sequencing results of the two families.

Proband 2 presented with typical ODDD. He had short palpebral fissures and epicanthal folds, as well as other typical ODDD facial features, such as hypoplastic alae nasi, a broad nasal bridge, prominent nasal tip and columella, and thin lips, accompanied by sparse hair. Limb abnormalities included bilateral soft tissue syndactyly of fingers 4–5 and bilateral syndactyly of toes 3–4, extending to the proximal phalanx on the left foot and the middle phalanx on the right foot. Dental anomalies, including microdontia, dental crowding, and enamel hypoplasia, were also observed (Figure 1A). As depicted in the pedigree (Figure 1B), both parents of Family 2 were clinically unaffected, suggesting a potential *de novo* mutation in Proband 2. A summary of clinical symptoms and cell culture status is also shown (Table 1). Diagnosis was based on at least two major criteria (facial dysmorphism, syndactyly, dental anomalies), confirmed by *GJA1* Sanger sequencing.

Flow cytometric analysis confirmed that both patient- and control-derived MSCs were positive for CD90, CD105, and CD73, and negative for CD45, consistent with the ISCT minimal criteria for MSCs (Supplementary Figure 1).

### Whole-genome sequencing and variant prioritization

WGS was performed on both probands. After filtering against dbSNP (v151), 1,000 Genomes (phase 3), and gnomAD (v3.1) with MAF <0.01, and excluding intergenic and intronic variants, candidate variants were prioritized by predicted functional impact (CADD >20) and OMIM disease-gene annotation. Notably, both probands presented with classic ODDD features, including craniofacial anomalies, syndactyly, and dental defects, which are highly characteristic of *GJA1*-related ODDD. Among the prioritized candidates, only the *GJA1* variants (c.253G>C in Family 1; c.605G>A in Family 2) segregated perfectly with the affected phenotype, while all other candidates were excluded due to lack of segregation. Given the consistent genotype–phenotype correlation, these two *GJA1* variants were selected for Sanger validation.

### Variant validation

Sanger sequencing validation was performed to confirm the candidate variants identified by WGS. In Family 1, a heterozygous missense variant, c.253G>C, in the *GJA1* gene was identified in the proband, her affected younger brother, and their mildly affected mother (presenting with bilateral brachydactyly of the fifth fingers), while it was absent in the unaffected father, confirming co-segregation with the phenotype. In Family 2, a distinct heterozygous missense variant, c.605G>A, in the *GJA1* gene was identified in the proband but was absent in both clinically healthy parents, indicating a *de novo* mutation. Representative Sanger sequencing chromatograms for the two families are shown in Figure 1C.

### RNA sequencing detection results

RNA sequencing (RNA-seq) was performed on dermal fibroblasts from two ODDD patients (P1 and P2) and MSCs from patient P1, with matched controls as baseline. KEGG and GO enrichment analyses were applied to the differentially expressed genes (Figure 2).

**FIGURE 2:**
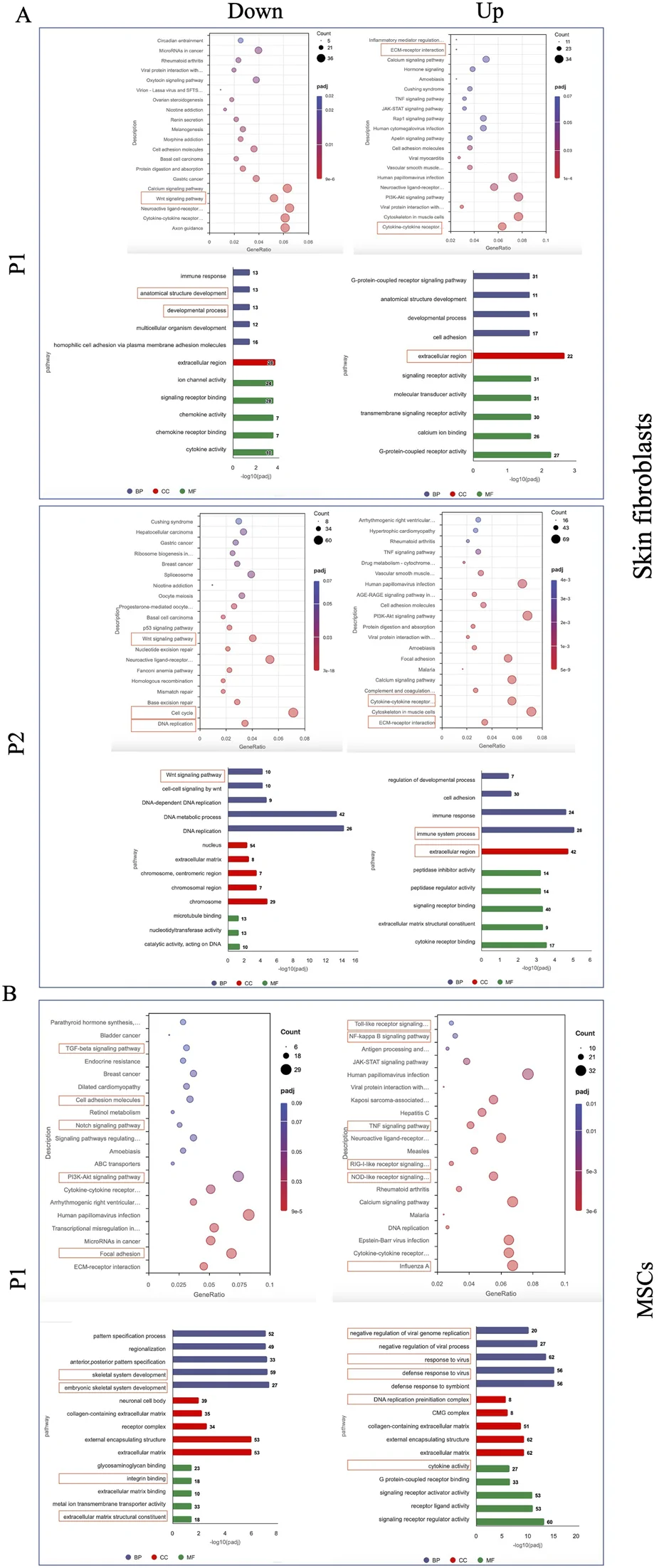
KEGG and GO enrichment analysis of differentially expressed genes in ODDD cells. **(A)** Skin fibroblasts from patient 1 and 2. **(B)** MSCs from patient 1. The gene sets of patients who were downregulated (Down) and upregulated (Up) compared to the normal control group.

The downregulated gene sets were predominantly enriched in the Wnt signaling pathway, ECM-receptor interaction, osteoblast differentiation, as well as DNA replication and cell cycle regulation. Conversely, the upregulated gene sets showed robust enrichment in the inflammatory response, cellular response to interleukin-1, cytokine-mediated signaling pathway, JAK-STAT signaling pathway, response to tumor necrosis factor (TNF), regulation of apoptotic process, and extracellular matrix (ECM) structural constituents and organization. The transcriptomic signature revealed by differential gene enrichment perfectly aligns with subsequent protein-level and cellular functional validation, constructing a complete evidence chain spanning transcriptional reprogramming, protein expression alteration and phenotypic dysfunction in ODDD cells.

### Wnt/β-catenin inhibition, inflammatory bias, and high MMP3 expression in cells from ODDD patients

To validate the transcriptomic findings at the transcriptional and translational levels, we performed RT-qPCR, Western blot, immunofluorescence, and ELISA assays on skin fibroblasts and MSCs derived from ODDD patients.

In fibroblasts and MSCs (Figure 3A), RT-qPCR results showed that *GJA1* mRNA expression was significantly downregulated in patient cells compared to the control group. Consistent with the suppression of the Wnt/β-catenin signaling pathway, mRNA levels of the classic Wnt target genes *AXIN2*, *CCND1*, *PCNA*, and *c-MYC* were significantly reduced in both patient fibroblasts and MSCs. Regarding the inflammatory profile, *IL-6* expression was significantly upregulated, while *CXCL8*(*IL-8*) was paradoxically and significantly downregulated; simultaneously, expression of the interferon-stimulated genes *MX1* and *IFIT1* was significantly elevated. Additionally, *MMP3* mRNA expression was significantly increased in patient cells. Western blot analysis further confirmed these molecular changes at the protein level (Figure 3B). In patient fibroblasts and MSCs, the protein levels of Cx43, β-catenin, and IκBα were all significantly reduced; conversely, the ratio of phosphorylated p65 to total p65 (P-p65/p65) was elevated, suggesting activation of the NF-κB pathway, and MMP3 protein expression was significantly upregulated. Immunofluorescence staining visually confirmed the upregulation of MMP3 (green fluorescence) and downregulation of β-catenin (red fluorescence) in patient fibroblasts and MSCs (Figure 3C). Finally, ELISA results showed that IL-6 secretion levels were significantly increased, while IL-8 secretion levels were significantly decreased in the culture supernatants of patient cells, consistent with the RT-qPCR results (Figure 3D). In summary, these results confirm a consistent molecular landscape in ODDD patient cells, characterized by Wnt/β-catenin inhibition, NF-κB activation, a specific inflammatory bias, and upregulation of MMP3.

**FIGURE 3:**
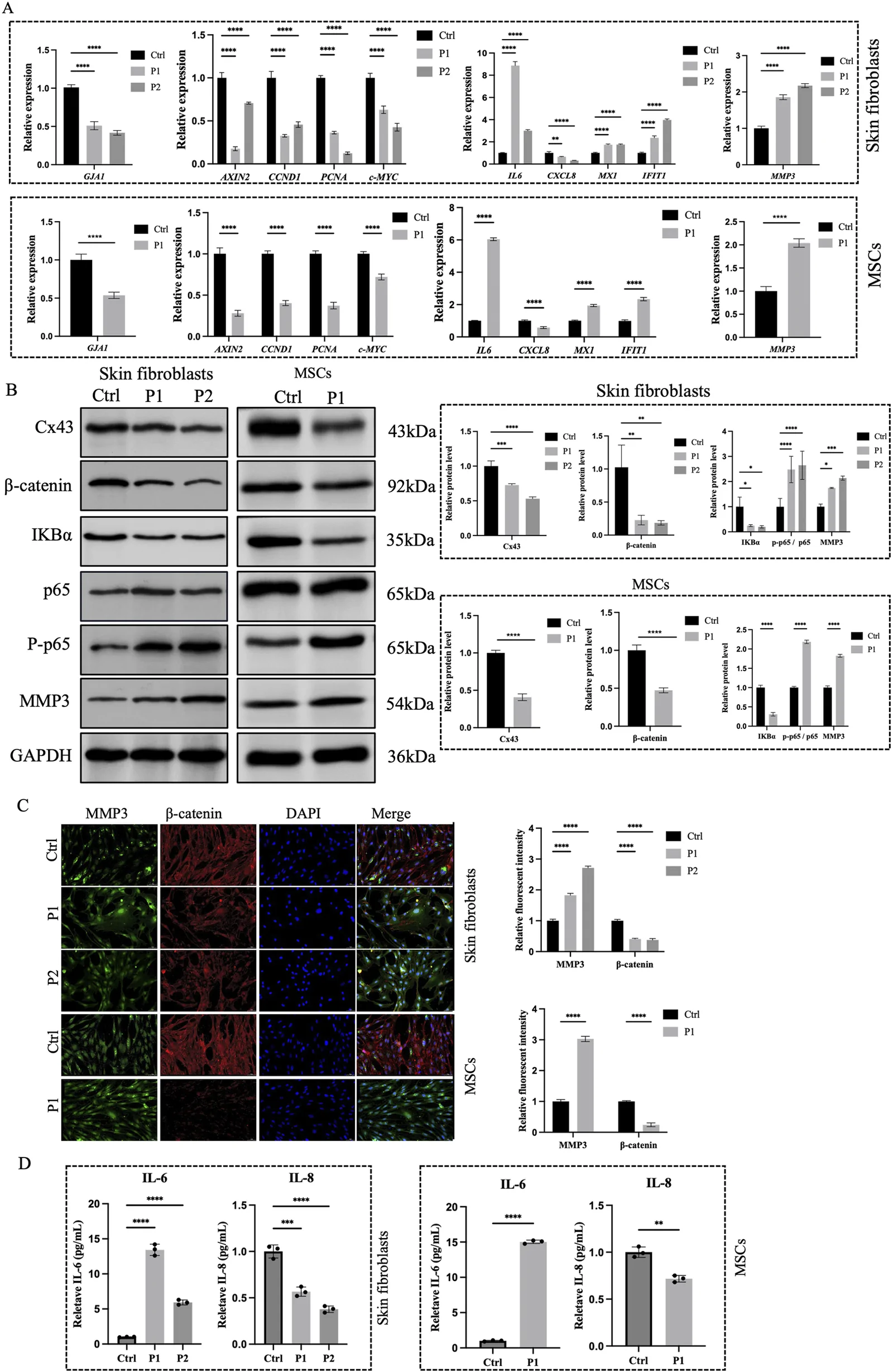
Validation of Wnt suppression, inflammatory bias, and MMP3 upregulation in ODDD cells. **(A)** Analysis of the relative expression levels by RT-qPCR of *GJA1*, Wnt pathway target genes (*AXIN2*, *CCND1*, *PCNA*, *c-MYC*), genes associated with inflammation and innate immunity (*IL6*, *CXCL8*, *MX1*, *IFIT1*), and the matrix metalloproteinase *MMP3* in skin fibroblasts and MSCs, with expression levels normalized to *GAPDH*. **(B)** Representative Western blot images and quantitative analysis of Cx43, β-catenin, IκBα, p65, P-p65, and MMP3 in patient and control cells. **(C)** Immunofluorescence staining for MMP3 (green) and β-catenin (red) in skin fibroblasts and MSCs. Cell nuclei were counterstained with DAPI. Right: quantitative densitometric analysis. **(D)** ELISA quantification of IL-6 and IL-8 secretion in culture supernatants from patient and control cells. Data are shown as mean ± SD, with all individual data points. Normal controls: n = 3 independent donors (biological replicates). Patient groups: n = 1–2 probands with three technical replicates from different passages per sample. Patient vs. control comparisons used an unpaired two-tailed t-test (technical replicates vs. biological replicates), following Botta et al. (2023, EMBO J). Three-group comparisons used one-way ANOVA with Tukey’s *post hoc* test; two-group comparisons used an unpaired t-test. Within-patient comparisons used paired t-tests. *p < 0.05, **p < 0.01, ***p < 0.001, and ****p < 0.0001.

### Decreased migration and proliferation capacities of patient-derived cells in ODDD

To assess the functional consequences of molecular alterations in ODDD patient cells, we evaluated the migration and proliferation capacities of patient-derived skin fibroblasts and mesenchymal stem cells (MSCs). Results from the Transwell migration assay showed that, compared with the normal control group (Ctrl), the number of cells crossing the basement membrane was significantly reduced in patient-derived skin fibroblasts (P1, P2) and mesenchymal stem cells (P1), indicating impaired migration capacity in patient cells (Figure 4A). Additionally, EdU incorporation assays revealed that the proportion of EdU positive cells in patient-derived skin fibroblasts and mesenchymal stem cells was significantly lower than that in the normal control group, suggesting that the proliferative capacity of patient cells was also markedly suppressed (Figure 4B). Consistent with these functional data, RNA-seq enrichment analysis revealed that downregulated genes in patient cells were significantly enriched in DNA replication and cell cycle regulation (Figure 2). Combined with the coordinated downregulation of *CCND1*, *PCNA*, and *c-MYC*—key regulators of the G1/S transition—these findings suggest that *GJA1* deficiency impairs G1/S progression and reduces S-phase entry. In summary, these functional assay results indicate that cells derived from ODDD patients exhibit dual defects in migration and proliferation.

**FIGURE 4:**
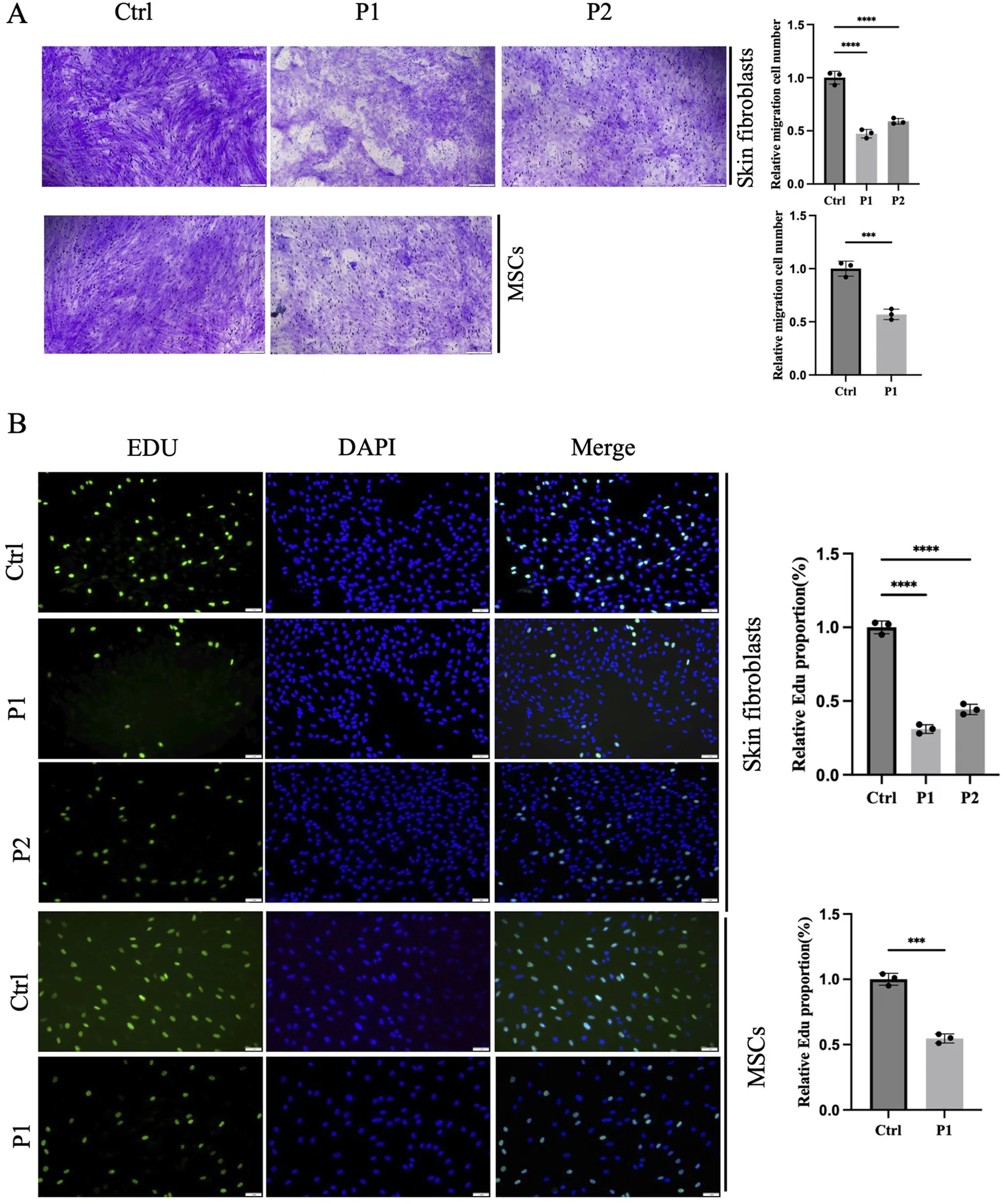
Impaired cell migration and proliferation in ODDD patients. **(A)** Transwell migration assay was used to assess the migration capacity of skin fibroblasts (top) and mesenchymal stem cells (bottom) from patients and healthy controls; the bar charts show quantitative analysis of the relative number of migrating cells. **(B)** EdU incorporation assay was used to assess the proliferative capacity of patient and normal control cells; the bar charts show a quantitative analysis of the relative proportion of EdU-positive cells (green: EdU; blue: DAPI). Data are shown as mean ± SD, with all individual data points. Normal controls: n = 3 independent donors (biological replicates). Patient groups: n = 1–2 probands with 3 technical replicates from different passages per sample. Patient vs. control comparisons used an unpaired two-tailed t-test (technical replicates vs. biological replicates), following Botta et al. (2023, EMBO J). Three-group comparisons used one-way ANOVA with Tukey’s *post hoc* test; two-group comparisons used an unpaired t-test. Within-patient comparisons used paired t-tests. *p < 0.05, **p < 0.01, ***p < 0.001, and ****p < 0.0001.

### GJA1 manipulation confirms its causal role in ODDD cell phenotypes

To determine whether *GJA1* deficiency is the fundamental driver of the aberrant phenotypes in ODDD, we performed siRNA-mediated knockdown of *GJA1* in normal cells (Ctrl + siGJA1) and overexpressed wild-type *GJA1* in patient cells (P1+OE-GJA1) to test whether these interventions could respectively mimic or rescue the pathological phenotypes. These molecular and functional analyses were conducted in both MSCs and FBs. Figure 5 displays the representative results from MSCs, while the corresponding data from FBs, which exhibited identical trends, are presented in Supplementary Figure 2. At the transcriptional level, RT-qPCR results (Figure 5A) confirmed that *GJA1* mRNA in normal cells was successfully downregulated to levels comparable to those in patient cells (P1+Vector), while P1+OE-GJA1 significantly restored *GJA1* expression close to the normal control levels (Ctrl + siNC). Correspondingly, *GJA1* knockdown in normal cells partially recapitulated the patient phenotype, including Wnt signaling deficiency (downregulation of *AXIN2*, *CCND1*, *PCNA*, *c-MYC*), inflammatory bias (*IL-6* upregulation, *CXCL8* downregulation, MX1/IFIT1 upregulation), and aberrant elevation of *MMP3*. Conversely, overexpression of wild-type GJA1 in patient cells substantially reversed these transcriptional alterations, bringing most gene expression levels closer to the normal ranges. At the protein level, Western blot analysis (Figure 5B) aligned with the qPCR findings. In the Ctrl + siGJA1 group, Cx43, β-catenin, and IκBα protein levels decreased, while the p-p65/p65 ratio and MMP3 protein levels increased, closely mimicking the patient (P1+Vector) profile. In contrast, the P1+OE-GJA1 group effectively rescued IκBα degradation, reduced p-p65 phosphorylation, and suppressed MMP3 expression. ELISA further confirmed that the aberrant secretion of IL-6 and IL-8 in culture supernatants followed the same trend of knockdown-induced stimulation and overexpression-mediated partial rescue (Figure 5C). Functionally, Transwell migration assays (Figure 5D) revealed that *GJA1* knockdown significantly impaired cell migration, whereas *GJA1* overexpression significantly restored the migration defects in patient cells. In assessments of multilineage differentiation potential, patient cells showed distinct defects compared to controls, including compromised osteogenic capacity (reduced ALP staining and Alizarin Red S mineralization, Figures 5E,F) and aberrantly enhanced adipogenic potential (increased Oil Red O staining, Figure 5G). Knockdown of *GJA1* in normal cells shifted their osteogenic/adipogenic profile towards the patient phenotype, while *GJA1* overexpression in patient cells partially restored osteogenic differentiation and suppressed the aberrantly elevated adipogenic differentiation.

**FIGURE 5:**
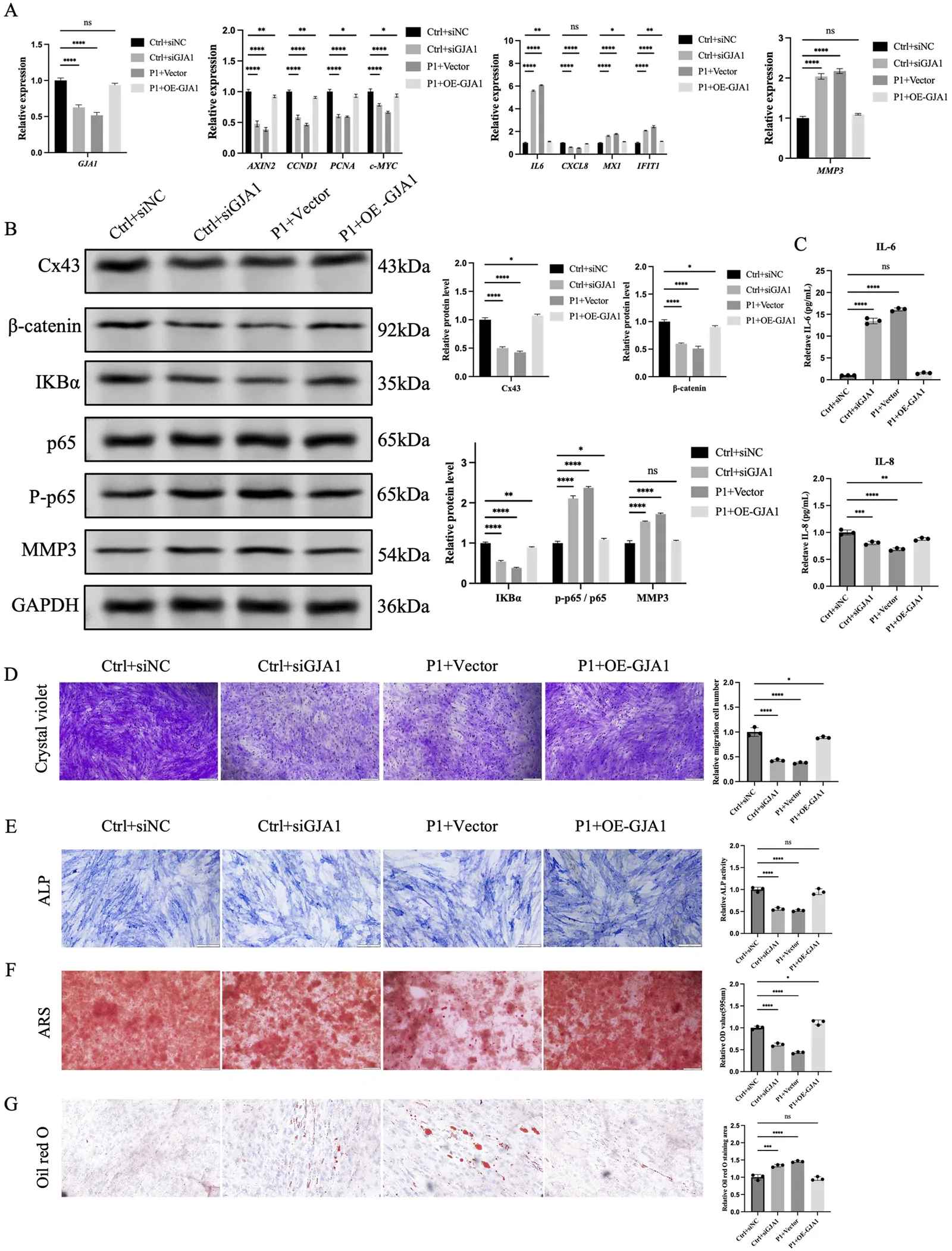
*GJA1* knockdown mimics ODDD phenotypes, while *GJA1* overexpression rescues molecular and functional defects in patient cells. **(A)** Relative mRNA expression levels of *GJA1*, Wnt targets (*AXIN2*, *CCND1*, *PCNA*, *c-MYC*), inflammatory and immune-related genes (*IL6*, *CXCL8*, *MX1*, *IFIT1*), and *MMP3* in the four treatment groups (Ctrl + siNC, Ctrl + siGJA1, P1+Vector, P1+OE-GJA1) determined by RT-qPCR. **(B)** Representative Western blot images and quantitative analysis of Cx43, β-catenin, IκBα, p65, P-p65, MMP3, and GAPDH (internal control) in the four groups. **(C)** ELISA quantification of IL-6 and IL-8 secretion levels in the culture supernatants from the four groups. **(D)** Representative images of Transwell migration assays and quantitative analysis of relative migrated cell numbers in the four groups. **(E)** Alkaline phosphatase (ALP) staining and relative activity quantification in the four groups. **(F)** Alizarin Red S (ARS) staining and relative mineralization quantification in the four groups. **(G)** Oil Red O staining and relative adipogenic capacity quantification in the four groups. Data are shown as mean ± SD, with all individual data points. Normal controls: n = 3 independent donors (biological replicates). Patient groups: n = 1–2 probands with 3 technical replicates from different passages per sample. Patient vs. control comparisons used an unpaired two-tailed t-test (technical replicates vs. biological replicates), following Botta et al. (2023, EMBO J). Three-group comparisons used one-way ANOVA with Tukey’s *post hoc* test; two-group comparisons used an unpaired t-test. Within-patient comparisons used paired t-tests. *p < 0.05, **p < 0.01, ***p < 0.001, and ****p < 0.0001.

Collectively, these bidirectional genetic interventions provide compelling evidence that *GJA1* deficiency serves as the core upstream driver triggering Wnt/β-catenin suppression, NF-κB/inflammatory bias activation, triggering aberrant MMP3 expression, and altered migration/multilineage differentiation capacities in ODDD cells.

### c-MYC is a key regulator of CXCL8 and MMP3 expression

To directly test whether *c-MYC* mediates the inflammatory bias and *MMP3* dysregulation, we performed *c-MYC* knockdown in normal MSCs and *c-MYC* overexpression in patient-derived MSCs (P1), with four experimental groups: Ctrl + siNC, Ctrl + si-cMYC, P1+Vector, and P1+c-MYC-OE. As shown in Figure 6A, siRNA-mediated knockdown of *c-MYC* in normal MSCs reduced *c-MYC* mRNA to a level comparable to that in patient cells (P1+Vector), confirming successful mimicry of the patient *c-MYC* deficiency. This *c-MYC* reduction was accompanied by a significant decrease in *CXCL8* and a marked increase in *MMP3*, reproducing the transcriptional signature of patient cells. Conversely, overexpression of c-MYC in patient MSCs restored *c-MYC* mRNA to a level similar to that of normal controls, which significantly rescued the *CXCL8* suppression and suppressed the aberrant *MMP3* upregulation. ELISA further confirmed these findings at the protein secretion level (Figure 6B). IL-8 secretion was significantly reduced in Ctrl + si-cMYC cells, to an extent comparable to that in P1+Vector patient cells, while c-MYC overexpression in patient cells significantly restored IL-8 secretion. Together, these gain- and loss-of-function experiments establish c-MYC as a necessary and sufficient regulator of CXCL8 and MMP3 downstream of the GJA1/Wnt/β-catenin axis.

**FIGURE 6:**
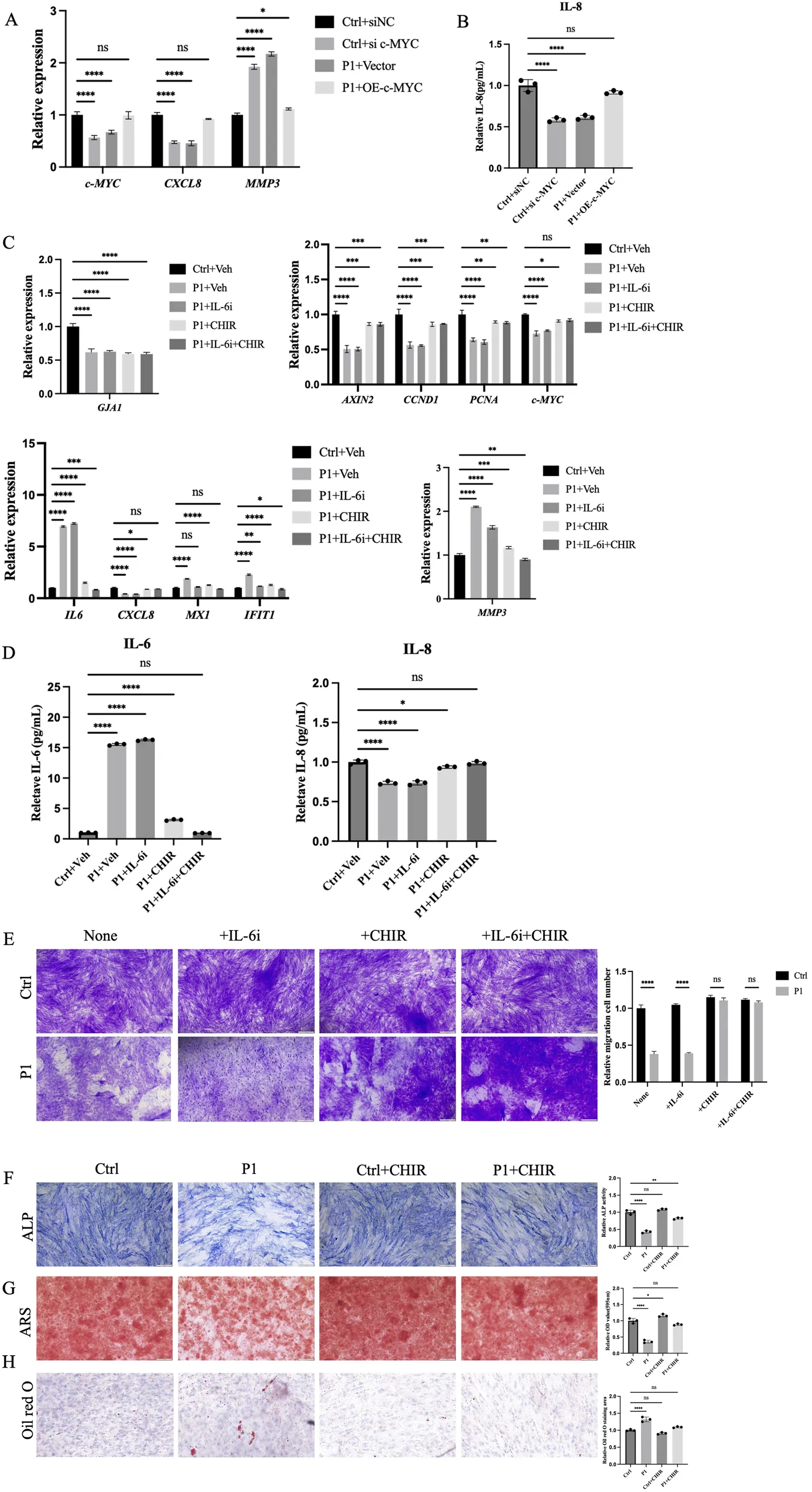
c-MYC mediates CXCL8 and MMP3 expression, and pharmacological interventions reveal the central role of Wnt/β-catenin. **(A)** RT-qPCR analysis of c-MYC, CXCL8, MMP3, and GJA1 in Ctrl + siNC, Ctrl + si-cMYC, P1+Vector, and P1+c-MYC-OE groups. **(B)** ELISA quantification of IL-8 secretion in the same four groups. Data are mean ± SD. Normal controls: n = 3 donors; patient groups: n = 1 donor (P1) with three technical replicates. Individual data points are shown. *P < 0.05, **P < 0.01, ***P < 0.001. **(C)** RT-qPCR analysis of GJA1, Wnt target genes (AXIN2, CCND1, PCNA, c-MYC), inflammatory genes (IL6, CXCL8, MX1, IFIT1), and MMP3 in five groups: Ctrl + Veh, P1+Veh, P1+IL-6i, P1+CHIR, and P1+IL-6i + CHIR. **(D)** ELISA quantification of IL-6 and IL-8 secretion in the five groups. **(E)** Transwell migration assays. **(F–H)** Osteogenic and adipogenic differentiation (ALP, Alizarin Red S, Oil Red O). Data are shown as mean ± SD, with all individual data points. Normal controls: n = 3 independent donors (biological replicates). Patient groups: n = 1–2 probands with three technical replicates from different passages per sample. Patient vs. control comparisons used an unpaired two-tailed t-test (technical replicates vs. biological replicates), following Botta et al. (2023, EMBO J). Three-group comparisons used one-way ANOVA with Tukey’s *post hoc* test; two-group comparisons used an unpaired t-test. Within-patient comparisons used paired t-tests. *p < 0.05, **p < 0.01, ***p < 0.001, and ****p < 0.0001.

### Pharmacological interventions validate the Wnt/β-catenin pathway as a core hub in rescuing ODDD cellular phenotypes

To validate the hierarchical relationship of downstream signaling cascades, patient-derived MSCs were treated with the Wnt/β-catenin pathway activator CHIR99021 and IL-6 neutralizing antibody (IL-6i), either alone or in combination. RT-qPCR results (Figure 6C) showed that drug treatments did not alter the expression of variant *GJA1* in patient cells. For Wnt target genes, CHIR alone or in combination significantly upregulated *AXIN2*, *CCND1*, *PCNA*, and *c-MYC* in patient cells, whereas IL-6i alone showed no rescue effect. In the inflammatory signature, the abnormally elevated *IL6* was suppressed by CHIR or combined treatment; conversely, the paradoxically downregulated *CXCL8* was significantly restored only in the CHIR or combined groups. Furthermore, although IL-6i effectively downregulated *MX1* and I*FIT1*, it failed to suppress the aberrantly upregulated MMP3, which was effectively inhibited by CHIR. ELISA results confirmed these trends at the protein secretion level (Figure 6D). CHIR or combined treatment significantly reduced IL-6 secretion and restored IL-8 secretion in patient cells, whereas IL-6i alone failed to rescue either. Regarding cellular functional phenotypes, Transwell migration assays (8 groups, Figure 6E) revealed that CHIR or combined treatment significantly rescued the impaired migration of patient cells, while IL-6i alone was largely ineffective. In the assessment of differentiation potential (4 groups: Ctrl, P1, Ctrl + CHIR, P1+CHIR), patient cells (P1) exhibited compromised osteogenic capacity (reduced ALP activity and Alizarin Red S staining) and enhanced adipogenic capacity (increased Oil Red O staining). Treatment with the Wnt agonist CHIR (P1+CHIR) effectively restored osteogenic differentiation and significantly suppressed the abnormally enhanced adipogenic differentiation in patient cells (Figures 6F–H).

Collectively, these pharmacological interventions establish the Wnt/β-catenin pathway as the core upstream “switch” in ODDD; activating Wnt signaling rescues molecular defects, migration impairments, and osteogenic/adipogenic imbalances. Hierarchical comparison of pharmacological treatments revealed clear signaling cascade priority: isolated IL-6 neutralization only partially alleviated interferon-stimulated gene expression but failed to restore upstream Wnt/c-MYC activity, thus unable to rescue CXCL8 suppression or reduce MMP3 overexpression. In contrast, the Wnt/β-catenin pathway activator CHIR99021 acted at the core upstream hub to recover c-MYC expression, reverse inflammatory bias, inhibit extracellular matrix (ECM) degradation, and restore defective migration and osteogenic differentiation. Combined CHIR99021 and IL-6 inhibitor treatment exerted enhanced rescue effects by simultaneously targeting core pathway dysfunction and downstream inflammatory overactivation.

## Discussion

### Clinical heterogeneity and pathogenic variants of ODDD

ODDD is a rare autosomal dominant disorder primarily caused by variants in the *GJA1* gene, which encodes the gap junction protein connexin 43 (Cx43) (Laird, 2006). In this study, two families exhibited typical ODDD features, including craniofacial anomalies, syndactyly, and dental defects (Paznekas et al., 2003). Notably, we observed significant intra-familial phenotypic variability in Family 1, where the mother only exhibited mild bilateral brachydactyly while her children showed full penetrance. Previous studies using mutant mouse models have confirmed that *GJA1* variants cause extensive phenotypic variability, which aligns well with our observations (Flenniken et al., 2005; Dobrowolski et al., 2008). The variant in Family two was identified as a *de novo* event. Both variants likely disrupt the conserved structural domains of Cx43, impairing its trafficking and gap junction assembly (Kelly et al., 2016).

### GJA1 deficiency leads to central suppression of the Wnt/β-catenin pathway

A core finding of our study is the profound suppression of the Wnt/β-catenin signaling pathway in patient-derived dermal fibroblasts and MSCs. Transcriptomic profiling and RT-qPCR confirmed the marked downregulation of canonical Wnt targets *AXIN2*, *CCND1*, *PCNA*, and the critical transcriptional co-activator *c-MYC*. The Wnt/β-catenin signaling pathway is a well-established master regulator of skeletal development and cell fate determination (Baron and Kneissel, 2013). Cx43 interacts physically with β-catenin through its C-terminal domain (Spagnol et al., 2018). Thus, loss of functional Cx43 may perturb β-catenin-associated signaling and contribute to reduced Wnt transcriptional activity (Cai et al., 2026; Moorer et al., 2017). The downregulation of CCND1 and PCNA directly contributed to the impaired proliferation capacity of patient cells, while the loss of c-MYC acted as a pivotal switch for downstream phenotypic shifts. Notably, previous studies on ODDD have primarily focused on gap junction defects, impaired cell proliferation/migration, and delayed osteogenic differentiation (Smyth and Shaw, 2013; Martins-Marques et al., 2019; Chaible et al., 2011; McLachlan et al., 2008). However, the upstream signaling hubs and microenvironmental changes driving these pleiotropic phenotypes remained elusive. Our study breaks new ground by establishing that *GJA1* deficiency acts as a core upstream switch that triggers a global suppression of the Wnt/β-catenin signaling axis. Crucially, we unveiled a novel ‘specific inflammatory bias’ mediated by the loss of c-MYC, providing a direct mechanistic explanation for the paradoxical inflammatory signature in ODDD cells.

### Loss of c-MYC drives inflammatory bias and MMP3 derepression

Strikingly, we uncovered a specific and paradoxical inflammatory bias characterized by NF-κB activation (elevated p-p65/p65 ratio), leading to robust IL-6 upregulation, accompanied by a significant downregulation of CXCL8 (IL-8). Emerging evidence has demonstrated a complex crosstalk between the Wnt/β-catenin pathway and NF-κB-mediated inflammatory responses (Oeckinghaus et al., 2011). The loss of the Wnt downstream target c-MYC mechanistically explains the uncoupling of IL-6 and CXCL8 expression. Previous studies have implicated c-MYC in the regulation of CXCL8 expression, including its association with the CXCL8 promoter (Cheng et al., 2025). In our ODDD cells, reduced c-MYC expression coincided with marked CXCL8 suppression despite activation of NF-κB. These findings, together with our gain- and loss-of-function experiments, support a role for c-MYC in CXCL8 regulation downstream of the GJA1/Wnt/β-catenin axis. In our ODDD cell model, reduced Wnt/β-catenin activity was accompanied by increased MMP3 expression. Previous studies have shown that Wnt/β-catenin signaling can directly regulate MMP3 transcription in a cell-type- and context-dependent manner (Blavier et al., 2010). Consistent with a regulatory relationship between these pathways, our genetic and pharmacological experiments showed that restoration of Wnt/β-catenin signaling was associated with suppression of MMP3 in ODDD cells. However, the precise transcriptional mechanism linking Wnt/β-catenin signaling to MMP3 regulation in ODDD remains to be determined. The causal role of c-MYC was directly substantiated by our gain- and loss-of-function experiments, demonstrating that c-MYC contributes to the regulation of CXCL8 and MMP3 expression independently of upstream Wnt activation. MMP3 is an important matrix-degrading enzyme involved in ECM remodeling (Sbardella et al., 2012). Whether increased MMP3 contributes directly to the syndactyly phenotype in ODDD remains speculative and requires *in vivo* validation. In addition, the significant upregulation of the interferon-stimulated genes MX1 and IFIT1 was consistent with activation of interferon-associated transcriptional responses in ODDD cells (Ivashkiv and Donlin, 2014).

### The Wnt/c-MYC axis determines the osteogenic-adipogenic balance of MSCs

The multilineage differentiation imbalance in patient MSCs is another significant functional finding. Patient cells exhibited compromised osteogenic capacity but enhanced adipogenic potential. This phenotype perfectly aligns with the classical biological functions of the Wnt/β-catenin pathway—while maintaining MSC stemness and promoting osteogenesis, Wnt signaling generally suppresses adipocyte differentiation (Ross et al., 2000). In our ODDD cell model, the loss of Wnt signaling caused by the *GJA1* variant tilts the lineage commitment balance toward adipogenesis, which also explains the potential pathological basis of bone hypoplasia in ODDD patients from a cellular differentiation perspective.

### Genetic and pharmacological interventions reveal the Wnt pathway as the core upstream switch

To confirm that these pathological phenotypes are directly driven by *GJA1* deficiency, we performed bidirectional genetic interventions. SiRNA-mediated knockdown of *GJA1* in normal cells faithfully recapitulated the patient phenotypes—Wnt suppression, inflammatory bias, *MMP3* upregulation, and impaired migration/osteogenesis. Conversely, wild-type *GJA1* overexpression in patient cells significantly reversed these defects. Pharmacologically, the Wnt/β-catenin pathway activator Pharmacological activation of Wnt/β-catenin signaling with CHIR99021 restored c-MYC expression and was accompanied by recovery of *CXCL8*, suppression of *MMP3*, and improved osteogenic differentiation in our ODDD cells. These findings are consistent with previous evidence that CHIR99021 activates canonical Wnt signaling and promotes osteogenesis (Wang et al., 2022). In contrast, IL-6 neutralizing antibody (IL-6i) only attenuated downstream interferon responses but failed to restore c-MYC, rescue CXCL8, or suppress MMP3. This comparison strongly establishes that the Wnt/β-catenin pathway serves as the master upstream switch in regulating ODDD cellular phenotypes, highlighting that targeting Wnt signaling is more promising than merely targeting downstream inflammation.

### Limitations, conclusion, and therapeutic prospects

Despite the comprehensive nature of our study, some limitations should be noted. First, Cx43 is broadly expressed in the heart, central nervous system, and skin, and previous reports have documented cardiac arrhythmias, leukodystrophy, and palmoplantar keratoderma in patients carrying certain *GJA1* variants. Although the two families in this study primarily exhibit typical skeletal, dental, and digital defects, long-term follow-up for neurological and cardiac assessments is warranted to fully evaluate the potential multi-organ involvement. Additionally, our current conclusions rely primarily on *in vitro* patient-derived cell models, and future validation using *in vivo* animal models, such as *GJA1* variant mice, is required. Furthermore, the RNA-seq analysis of patient fibroblasts (n = 2) and MSCs (n = 1) is limited by small sample size, and these transcriptomic findings should be considered exploratory. Finally, although our gain- and loss-of-function experiments established c-MYC as a necessary and sufficient regulator of *CXCL8* and *MMP3*, we were unable to perform promoter-reporter or ChIP-based analyses to directly demonstrate c-MYC binding to the CXCL8 or MMP3 promoters. This limitation was due to the extremely limited number of patient-derived primary cells, particularly bone-derived MSCs from pediatric patients. Future studies with expanded patient-derived cell resources will be needed to directly validate this transcriptional mechanism. In conclusion, our study delineates a novel pathogenic network wherein *GJA1* dysfunction is associated with suppression of the Wnt/c-MYC axis, which in turn drives NF-κB-mediated inflammatory bias and *MMP3*-mediated ECM degradation. Our findings identify the restoration of Wnt signaling as a potent and promising therapeutic strategy for ODDD.

Another limitation concerns the statistical framework. Because ODDD is ultra-rare, fibroblasts were obtained from only two patients and MSCs from only one patient. For each patient sample, experiments were performed using at least three independent technical replicates (separately cultured passages). For comparisons between patient and control groups, the technical replicates of the patient sample were compared against the biological replicates of three healthy donors using unpaired two-tailed Student’s t-tests. This approach follows the established framework for statistical analysis in rare disease studies (Botta et al., 2023), but we acknowledge that technical replicates from a single donor are not equivalent to independent biological replicates. Therefore, these comparisons should be interpreted with caution, and the findings should be considered exploratory and proof-of-concept rather than definitive. Future studies with additional ODDD patients and independent MSC donors are needed to validate the generalizability of our results.

## Conclusion

In summary, this work establishes a novel pathogenic cascade for ODDD driven by *GJA1* pathogenic variants. Mechanistically, loss-of-function *GJA1* variants trigger global suppression of the Wnt/c-MYC signaling axis, which simultaneously initiates two parallel pathological branches: MMP3-mediated excessive extracellular matrix degradation, and a unique c-MYC-dependent inflammatory bias featuring NF-κB-dependent IL-6 elevation and paradoxical CXCL8 downregulation. From a translational perspective, pharmacological activation of Wnt signaling comprehensively rescues defective cell proliferation, migration and unbalanced mesenchymal lineage differentiation, whereas isolated IL-6 blockade only relieves partial downstream inflammatory readouts without correcting core upstream pathway defects. Our findings highlight Wnt/c-MYC restoration as a promising fundamental therapeutic strategy for oculodentodigital dysplasia.

## Data Availability

The raw data generated in this study contain identifiable information from human participants (patients and their family members). Due to ethical restrictions and participant privacy, the raw data cannot be made publicly available. However, anonymized datasets supporting the conclusions of this article will be made available by the corresponding author upon reasonable request, subject to institutional ethical approval.
